# Magnetic Position System Design Method Applied to Three-Axis Joystick Motion Tracking

**DOI:** 10.3390/s20236873

**Published:** 2020-12-01

**Authors:** Perla Malagò, Florian Slanovc, Stefan Herzog, Stefano Lumetti, Thomas Schaden, Andrea Pellegrinetti, Mohssen Moridi, Claas Abert, Dieter Suess, Michael Ortner

**Affiliations:** 1 Silicon Austria Labs GmbH, Sensor Systems, Europastraße 12, 9524 Villach, Austria; Stefano.Lumetti@silicon-austria.com (S.L.); Mohssen.Moridi@silicon-austria.com (M.M.); 2 University of Vienna, Physics of Functional Materials, Boltzmanngasse 5, 1090 Vienna, Austria; florian.slanovc@univie.ac.at (F.S.); claas.abert@univie.ac.at (C.A.); dieter.suess@univie.ac.at (D.S.); 3 ZF Friedrichshafen AG, Graf-von-Soden-Platz 1, 88046 Friedrichshafen, Germany; stefan.herzog3@zf.com (S.H.); Thomas.Schaden@zf.com (T.S.); 4 ZF Padova S.r.l., Marine and Special Driveline Technology, Via S. Andrea, 16, 38062 Arco (TN), Italy; andrea.pellegrinetti@zf.com; 5 University of Vienna Research Platform MMM Mathematics-Magnetism-Materials, Oskar-Morgenstern-Platz 1, 1090 Vienna, Austria

**Keywords:** magnetic position sensor systems, computational magnetism, magnet system design, analytical method, magnetic joystick, python

## Abstract

This manuscript discusses the difficulties with magnetic position and orientation (MPO) system design and proposes a general method for finding optimal layouts. The formalism introduces a system quality measure through state separation and reduces the question “How to design an MPO system?” to a global optimization problem. The latter is then solved by combining differential evolution algorithms with magnet shape variation based on analytical computations of the field. The proposed formalism is then applied to study possible realizations of continuous three-axis joystick motion tracking, realized with just a single magnet and a single 3D magnetic field sensor. The computations show that this is possible when a specific design condition is fulfilled and that large state separations as high as $1\;\mathrm{mT}{/}^{\circ }$ can be achieved under realistic conditions. Finally, a comparison to state-of-the-art design methods is drawn, computation accuracy is reviewed critically, and an experimental validation is presented.

## 1. Introduction

Magnetic position and orientation (MPO) detection systems determine the relative motion between permanent magnets and magnetic field sensors by measuring the modulation of the magnetic field. Such systems offer many advantages like robustness against dirt and temperature, long lifetimes ensured by contactless operation, as well as high resolutions at low cost and low power operation [1,2]. Multiple applications are treated in the literature, including proximity detection, linear motion systems [3,4], angle and rotation sensing [5,6], encoders [7], and more complex forms like motion tracking of six degrees of freedom (DoFs) [8,9]. Nowadays, more than one hundred MPO system applications exist in the automotive sector alone, including gas and brake pedals, gear shifts, indicator levers, side mirror position, wheel speed, and anti-lock braking system (ABS) sensors [10].

A position system of specific interest is the three-axis joystick, which combines regular 2D joystick motion with rotation of the lever about its own axis. This concept is commonly employed for control elements in the automotive [11,12], nautical [13,14], medical [15], and aerospace [16] domains as well as for consumer electronics applications like arcade sticks [17] and closed-circuit television (CCTV) steering [18]. State-of-the-art magnetic implementations combine two MPO systems, namely a 2D joystick and a rotation sensor [11,13]. The latter must be integrated into the movable joystick shaft, thereby making it difficult to mechanically manufacture this type of implementation. Recent proposals show that special cases of three-axis joystick motion tracking can be realized with a limited number of DoFs in the form of an MPO system with only a single magnet and a single 3D sensor [19,20,21]. Thanks to their simplified construction, such devices are highly cost-efficient, which is a critical aspect for industrial applications, as is also shown by recent sensor and magnetic material developments [22,23].

Readout of an MPO system requires reconstruction of the magnet position from the sensor outputs. This is closely related to the magnetostatic inverse problem that is often mathematically ill-posed and computationally demanding [24,25]. One of the biggest challenges is to solve the inverse problem within milliseconds in order to enable real-time measurement and interaction. State-of-the-art systems address this problem by approximating the field with simple harmonics that can be easily inverted [3,4]. Several field-shaping proposals improve this approach by engineering fields with specific desired forms through multi-magnet arrangements [26], shimming techniques, or complex magnet shapes with inhomogeneous magnetization [27,28]. When more than two DoFs and more complex motions are involved, a direct numerical inversion of 3D approximations of the magnetic field seems to be the only viable solution. Such approximations are based on analytical solutions of permanent magnet problems [8], dipole approximations [9], pre-computed solutions, or simple look-up tables [21] to achieve the necessary computation times for fast inversion.

While sophisticated techniques exist to solve the inverse problem, there is no patented procedure on how to layout an MPO system, i.e., how to generally arrange magnets and sensors in order to realize the desired motion parameters of interest in the best possible way. For this, state-of-the-art implementations rely mostly on experience and educated guesses combined with point-wise finite element (FE) simulations for layout testing and optimization, an approach which is limited by the intrinsically long computation times involved. It is the aim of this paper to overcome these limitations by using computationally efficient analytical methods to find possible MPO system realizations and optimal layouts.

The paper structure is as follows: In Section 2.1, a general formalism is introduced which describes the conditions for a feasible and optimal MPO system layout. This formalism is compared to state-of-the-art field shaping methods in Section 2.2. A description of the three-axis-joystick is then given in Section 2.3. Magnetic field computations based on analytical solutions are discussed in Section 2.4, and differential evolution is suggested in Section 2.5 as a suitable global optimization method. The formalism is then applied to the three-axis-joystick problem, demonstrating under which conditions a continuous three-axis motion can be realized (Section 3.1) and how to find optimal layouts (Section 3.2). Two realistic and optimized layouts are proposed in Section 3.3. Finally, a comparison with an experiment is performed in Section 3.4.

## 2. Methods

### 2.1. General Formalism

This section introduces a general formalism for the design and optimization of MPO sensor systems, starting with the following relevant quantities:

- the observables of interest α∈P, where *P* is the parameter space of interest. Typical observables can be the lever position in a gear shift or the tilt angle of a joystick. The parameter space *P* then simply corresponds to the allowed range of mechanical motion.
- the system design parameters s∈S describe a specific MPO system implementation attempting to realize α. The system parameters include all quantities that can be varied within an allowed system parameter space *S* in a design process. They include magnet and sensor choice and placement within the system, component geometries, or material parameters. The parameter range *S* can be for instance the result of limited construction space.
- the sensor outputs $B\left(\alpha ,s\right)\in B_{\mathrm{space}}$ that correspond to single or multiple components of the magnetic field at the sensor positions. Although linear sensing technology is considered in this work, the formalism can be easily extended to include arbitrary sensor transfer functions. Hereafter, the sensor output and the magnetic field will be treated the same, and therefore, $B_{\mathrm{space}}$ will simply denote the set of all possible sensor outputs.
- a set of constraints C(s) that must be fulfilled in the design process. They can for example describe weighted sensing resolutions, maximal cost limitations, or the influence of system fabrication tolerances and external stray fields.

The central goal of MPO system design and optimization is to understand how well the observables of interest α can be determined from the sensor outputs B for a given system s and how to optimally design such a system when subjected to a limited system parameter range *S* and constraints C. It is critical to understand that the proposed formalism holds generally for any MPO system as the fundamental position detection limitation is given only by the field(s) at the sensor position(s). Any function composition f(B) including sensor transfer functions or combinations of different field components, like the $\arctan_{2}\left(B_{eve},B_{odd}\right)$ scheme in linear position systems [3], cannot improve system performance that is fundamentally limited by the magnetic field state density with respect to the natural sensor noise. In the case of the linear position system, for example, the goal is only to ease interpretation of the sensor output signal: the field ratio reduces the strong airgap dependence and the $\arctan_{2}$ function naturally stitches discontinuities and linearizes the output.

The fundamental requirement for a system s to realize the observable parameter space *P* is that each state α∈P is associated with a unique sensor output $B_{s}\in B_{\mathrm{space}}$. In other words, the magnetic field at sensor B(α,s) must be a bijective map between *P* and $B_{\mathrm{space}}$. Invertibility of the magnetic field on *P* is guaranteed when B is smooth and locally invertible for every α∈P and when $B_{\mathrm{space}}$ is simply connected. The smoothness of B can be assumed from the structure of the problem (smooth input variables and smooth fields of permanent magnets). Simple connectedness of $B_{\mathrm{space}}$, however, must always be checked [29]. A brief discussion of this is presented in Appendix A. In agreement with the inverse function theorem, local invertibility of a multivariate vector function is ensured when the Jacobian matrix has full rank. The Jacobian J is defined in the usual way:

(1) $$J_{ij}=\frac{\partial B_{i}\left(\alpha ,s\right)}{\partial \alpha_{j}}.$$

B is thus invertible on *P* for a specific s if

(2) detJ(α,s)≠0∀α∈P,

under the assumption that α and B have the same dimension. In the more general case, when the dimension of B is larger than the one of α, meaning there are more sensor outputs than observables of interest, then $B_{\mathrm{space}}$ is simply a manifold with similar dimension as α, embedded in a higher dimensional space, and the requirement (2) becomes

(3) $$\det\left(J{\left(\alpha ,s\right)}^{T}J\left(\alpha ,s\right)\right)\neq 0\;\;\forall \alpha \in P.$$

A system s is feasible (can in principle be realized) if Equation (3) can be fulfilled. Moreover, by means of Jacobian analysis, the density of states (DOS) denoted by *D* can be directly obtained as

(4) $$D\left(\alpha ,s\right)=\det{\left(J{\left(\alpha ,s\right)}^{T}J\left(\alpha ,s\right)\right)}^{-1/2}.$$

The inverse of the DOS corresponds to the state separation $\Delta p=D^{-1}$. Large state separations make it easier to detect different system states and, as such, Δp(α,s) provides an estimation of the quality of a specific MPO system implementation s. Hence, a quality factor *Q* can be introduced:

(5) $$Q\left(s\right)=\min_{\alpha \in P}\left(\Delta p(\alpha ,s)\right)\;,$$

as the minimal state separation (weakest link) for a given system s. Systems with large *Q*-factors are preferable from a technical point of view because their individual states are better separated—and therefore easier to detect—and distortions from external influences typically play a lesser role. For a given system parameter range *S* and set of constraints C, the best possible system $s_{\mathrm{opt}}$ is thus obtained as a result of the following optimization problem:

(6) $$s_{\mathrm{opt}}=\underset{s\in S\;:\;C(s)}{arg max}\left(Q\left(s\right)\right).$$

Equations (2)–(6) are the core instruments to answer the following questions: “How can an MPO system be realized?” and “What is the best possible realization?”. While state-of-the-art MPO design relies on educated guesses on which magnet-sensor arrangement can realize the desired observables of interest, Equation (6) simply reformulates this task as a global optimization problem.

**Figure 1:**
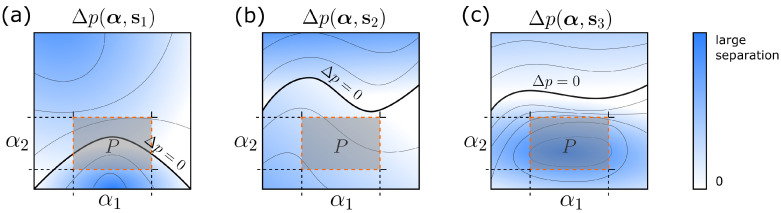
Schematic illustration of the state separation Δp as a function of the observables of interest α∈P for 3 different implementations: (**a**) System $s_{1}$ violates the feasibility criterion expressed by Equation (3). (**b**) System $s_{2}$ is a feasible implementation but with bad state separation. (**c**) System $s_{3}$ represents an optimal implementation that not only is feasible but also exhibits a large state separation.

To better understand the formalism, Figure 1 shows a sketch of the state separation for three different system implementations $s_{1}$, $s_{2}$, and $s_{3}$ together with the parameter space *P* of interest. Figure 1a represents a system where Equation (3) is violated. Figure 1b shows an implementation which is in principle possible (i.e., (3) is satisfied) but where state separations are bad (Equation (6) not fulfilled). Finally, Figure 1c represents an optimal solution satisfying Equation (6) with large state separations inside *P*.

### 2.2. Field Shaping and Shape Variation

The optimization problem (6) is reminiscent of inverse magnet design for field shaping [24,26]. There is however a crucial difference. While field shaping attempts to give the magnetic field a specific, favorable form (e.g., a linear component [26]) which can then be easily processed for readout, Equation (6) only aims to find the configuration with maximal state separation and relies as such on a more complex form of direct inversion for readout [8]. Field shaping thus requests preliminary knowledge of a target field, whereas the proposed MPO design method circumvents this requirement by simply looking at all possible solutions.

To solve the optimization problem (6), it seems nevertheless reasonable to extend field shaping techniques to MPO design. In this context, topology optimization using the adjoint method is able to address thousands of DoFs and to find optimal magnet forms ab initio [24,30,31,32]. However, remarkable results can already be achieved by variation of simple magnet shapes, as proposed in [26]. Comparison and discussion of the two methods are given in Appendix B.

In this paper, an extended version of the shape variation approach is chosen instead of complex procedures like the adjoint method for several reasons. First, the simple magnet forms are commercially available and cheap, while the slightly better performing but very complex magnet shapes that result from the adjoint method are hard to obtain and expensive in fabrication. In addition, the development effort required by shape variation is relatively small, whereas it can be demanding to formulate the complete MPO problem so that it can be treated with the adjoint method, which requires a priori calculation of an analytical variational derivation of the cost function. Complications may arise, for instance, when non-differentiable functions like min or max are involved.

Finally, it is worth noting that field shaping and the proposed MPO design should not be viewed as opposing strategies but rather as synergetic approaches. Indeed, MPO design by shape variation is limited to fewer DoFs and focuses on answering the question of how to realize a motion, which could, in turn, hint at potential target magnetic field shapes suitable for more sophisticated field shaping methods dealing with more DoFs and complex magnet forms.

### 2.3. The Three-Axis Joystick System

Here, the general formalism developed in Section 2.1 is applied to describe a specific MPO system of interest: a three-axis joystick in which the lever can be continuously tilted in two directions and rotated about its own axis. The objective is to resolve this three-axis motion by using only a single magnet and a single 3D magnetic field sensor. Previous works [19,20,21] focused on the extreme cost-efficiency of such an implementation in comparison to state-of-the-art solutions [11,13]. However, they only concentrated on a much simpler implementation with discrete tilt directions and tilt angles.

**Figure 2:**
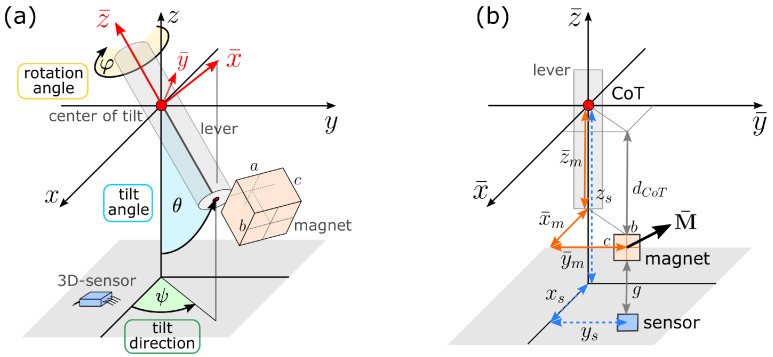
(**a**) Illustration of the magnetic joystick system with all its relevant components: the observables of interest are the three angles ψ, θ, and φ describing the joystick motion. The local coordinate system (red axes), fixed to the lever, is denoted by barred variables. (**b**) Sketch of the critical system parameters.

A convenient representation of the observables of interest describing the three-axis motion is given by the three angles ψ, θ, and φ sketched in Figure 2a: ψ is the azimuth angle corresponding to the lever tilt direction, θ is the polar angle indicating the amplitude of tilt, and φ is the rotation angle tracking the lever rotation about its own axis.

According to the general formalism, the observables of interest are thus α=(ψ,θ,φ). The parameter space *P* is defined by the corresponding allowed angle ranges, which—consistent with typical three-axis joystick motion—are chosen as $\psi \in {\left[0,360\right]}^{\circ }$, θ$\in [0,\theta_{\max}]$, and $\varphi \in [\varphi_{\min},\varphi_{\max}]$.

The MPO system is realized by fixing a permanent magnet at the bottom of the lever and by mounting a 3D magnetic field sensor directly below. This configuration implies that α and the sensor output B have the same dimension, which reduces the feasibility study to a simpler sign analysis through the use of Equation (2).

The magnet is defined in a local coordinate system (LCS) fixed to the lever which is denoted by barred symbols $\left(\bar{x},\bar{y},\bar{z}\right)$ and coincides with the global coordinates $\left(x,y,z\right)$ when α=0. A set of critical system parameters s is given by the following geometrical and physical quantities:

- the position of a 3D magnetic field sensor $r_{s}=\left(x_{s},y_{s},z_{s}\right)$. The sensor output is the magnetic field vector B.
- the magnet position ${\bar{r}}_{m}=\left({\bar{x}}_{m},{\bar{y}}_{m},{\bar{z}}_{m}\right)$ in the LCS. The lengths ${\bar{x}}_{m}$ and ${\bar{y}}_{m}$ indicate lateral displacement of the magnet from the lever axis, while ${\bar{z}}_{m}$ is the distance of the magnet from the center of tilt.
- the magnet magnetization vector $\bar{M}=\left({\bar{M}}_{x},{\bar{M}}_{y},{\bar{M}}_{z}\right)$ defined in the LCS, assuming uniform magnetization.
- the size of the magnet given by its side lengths (a,b,c), considering a cuboid magnet shape with orientation ${\bar{e}}_{i}^{m}$ in the LCS. The cuboid magnet shape is chosen for computational reasons; see Section 2.4.

These definitions naturally introduce an additional pair of critical parameters characteristic for such MPO systems, i.e., the airgap $g=z_{s}-{\bar{z}}_{m}$ between magnet and sensor as well as the magnet distance from the center of tilt $d_{CoT}={\bar{z}}_{m}-c/2$. Figure 2a,b shows a schematic of the magnetic joystick with all the corresponding relevant system parameters. The lever, the sensor, and all the other system component materials are chosen to be nonmagnetic (stainless steel, plastics, and silicon). The influences of sensor noise, possible magnetic shielding, external stray fields, or imperfect magnetization are neglected in this study.

The observables of interest α=(ψ,θ,φ) allow to infer the magnet position $r_{m}$ and orientation $e_{i}^{m}$ in the global coordinate system in terms of a rotation R(α) of the magnet position ${\bar{r}}_{m}$ and orientations ${\bar{e}}_{i}^{m}$ in the LCS,

(7) $$\begin{array}{cc} r_{m}\left(\alpha ,s\right) & =R\left(\alpha \right)\;{\bar{r}}_{m}\left(s\right), \end{array}$$

(8) $$\begin{array}{cc} e_{i}^{m}\left(\alpha ,s\right) & =R\left(\alpha \right)\;{\bar{e}}_{i}^{m}\left(s\right). \end{array}$$

A derivation of the rotation matrix R is reported in Appendix C. Assuming that the magnetic field can be computed in the LCS as $\bar{B}\left(\bar{r}\right)$, it is possible to determine the sensor output as:

(9) $$B\left(\alpha ,s\right)=R\left(\alpha \right)\bar{B}\left({\bar{r}}_{m}\left(s\right)-R^{-1}\left(\alpha \right)r_{s}\left(s\right)\right).$$

Equation (9) expresses the sensor output B in terms of both the observables of interest α and the system parameters s, which forms the basis for a further system analysis starting with Equation (1).

### 2.4. Magnetic Field Computation

Equation (9) requires the magnetic field $\bar{B}\left(\bar{r}\right)$ to be computed in the LCS. To this end, several viable options exist, the most commonly employed one being the FE method, as FE environments are readily available from multiple commercial and noncommercial sources [33,34,35,36], though the long computation times involved make such numerical approaches unpractical for dealing with the global multivariate optimization problem (6) that lies at the core of this paper.

As discussed in Section 2.2, the use of analytical solutions of permanent magnet problems [37,38] is envisaged in this work. Specifically, the field of cuboid magnets can be brought to a closed form [39,40], which enables a specifically fast computation of the sensor output. Moreover, cuboid-shaped magnets are commercially available off-the-shelf and their position and orientation can be defined very precisely when they are integrated into a mechanical setup, as opposed to spherical or cylindrical forms.

For implementation of the analytical formulas, the Magpylib Python package [41] is used, which is specially designed for dealing with MPO systems by integrating complex motions like those expressed by Equations (7)–(9). The analytical formulas are fully tested, vectorized, and optimized to ensure computational efficiency, achieving sub-microsecond computation times for calculation of the sensor output on standard x86 CPUs. In addition, a minimal development effort is required, since Magpylib enables system implementation with only a few lines of code, as demonstrated in Appendix D.

The analytical solution provides an excellent approximation of the magnetic field of realistic modern magnets despite neglecting demagnetization effects. A detailed discussion on the validity of this approximation is provided in Appendix E. In general, the error is less than 1% when $\mu_{r}<1.05$ (realistic for high-grade NdFeB, SmCo, or ferrite materials) and when the distance between sensor and magnet is of the same order or larger than the size of the magnet.

Finally, it must be noted that many MPO systems use soft magnetic materials as magnetic shields, flux guides, and concentrators or shimming elements [1,42]. However, not only can soft magnetic materials not be simulated analytically but also they bring a decisive disadvantage to MPO systems: they generate position-dependent external stray fields that cannot be compensated by differential measurement. The current trend in MPO systems is towards increased stray-field stability [6,43,44], and for this reason, soft magnetic components are generally avoided.

### 2.5. Optimization Algorithm

Equation (6) describes an optimization problem leading to the best possible MPO system layout $s_{\mathrm{opt}}$ that is subject to the constraints C and that realizes the parameters of interest α. The number of layout parameters and, therefore, the difficulty to solve the problem depend strongly on the complexity of the system. A single cuboid magnet already features 12 DoFs through its position, orientation, magnetization, and dimensions alone. For the typical 10–50 critical DoFs in MPO systems (see also the discussion in Appendix B), the differential evolution (DE) algorithm [45] is an excellent choice to solve such an optimization problem.

DE is a population-based evolutionary algorithm for the optimization of continuous variables in multidimensional spaces. Similar to genetic algorithms, it relies on an iterative process where a population is evolved by mutation, crossover, and selection to improve each generation. In contrast to genetic algorithms, however, DE avoids the harmful effect of mutation by carrying it out before the selection process [46].

The DE algorithm is especially well-suited to the MPO optimization problem (6) for several reasons. Firstly, the objective function is complex and its derivatives cannot be easily calculated, thereby favoring the use of such a black box optimization. Secondly, there can be multiple local optima which require the application of a global treatment. Finally, constraints can be easily included by nature of the algorithm, which adds to every new generation-only-allowed solutions.

DE for magnet shape variation relies heavily on the fast computation times provided by the analytical solutions proposed in Section 2.2 and Section 2.4 due to the large populations required for multivariate global optimization. In terms of computational efficiency, this approach can make optimal use of the computational resources on x86 type processors, as different field evaluations are completely independent of each other. The multiple field evaluations necessary to compute one objective function solution (α-grid spanning the space *P*) can be performed on the single instruction multiple data (SIMD) modules using the vectorized code from Magpylib, while the multiple objective function solutions (different values of s) required for constructing a population can be generated in parallel on separate cores. The SciPy [47] implementation of the DE algorithm provides automated multiprocessing and is used in this paper to perform the optimizations.

A similar computational setup was successfully used in the past for calibration of an MPO system [21]. The proposed implementation allows for reproducible and convergent parameter variations with up to several tens of parameters without resorting to extreme computation resources or distributed computation. A variation involving 20 system parameters and 460 field evaluations in the objective function was performed within 56 minutes and 44 seconds on an Intel^®^ Xeon^®^ Scalable Processor “Skylake” Gold 6126 (2.60 GHz, 12-Core Socket 3647, 19.25MB L3 Cache) running on 12 cores and converging within 2365 generations with population sizes of 2000 ($n_{\mathrm{pop}}=100$ in the algorithm). This corresponds to ∼639 field evaluations per millisecond, not counting the algorithm effort.

## 3. Results

### 3.1. Feasibility Analysis

A system s is defined as feasible if it can theoretically solve a given task, i.e., if there is a one-to-one correspondence between the mechanical states of interest α∈P and the sensor output B. Mathematically, this is expressed by Equation (2) for implementation of the three-axis joystick proposed in Section 2.3, where α and B are of similar dimensions.

A dipole moment is used as a magnetic field source for this feasibility analysis instead of a cuboid magnet. Since the dipole moment is the fundamental entity in magnetism and any magnetization distribution can be constructed from it by superposition, this approach provides a basic physical insight and is easily extended to finite-sized magnets. The magnetic field at the sensor location $r_{s}$ generated by a dipole moment m placed at the position $r_{m}$ is

(10) $$B\left(\alpha ,s\right)=\frac{\mu_{0}}{4\pi }\left(\frac{3(m\cdot r)r}{r^{5}}-\frac{m}{r^{3}}\right),$$

where $r\left(\alpha ,s\right)=r_{s}\left(s\right)-r_{m}\left(\alpha ,s\right)$ is the distance between the sensor and the magnetic dipole. The position $r_{m}$ and the orientation of the moment m are obtained for each mechanical state α through Equations (7) and (8), while the sensor position $r_{s}$ is fixed completely by the system parameters s.

**Figure 3:**
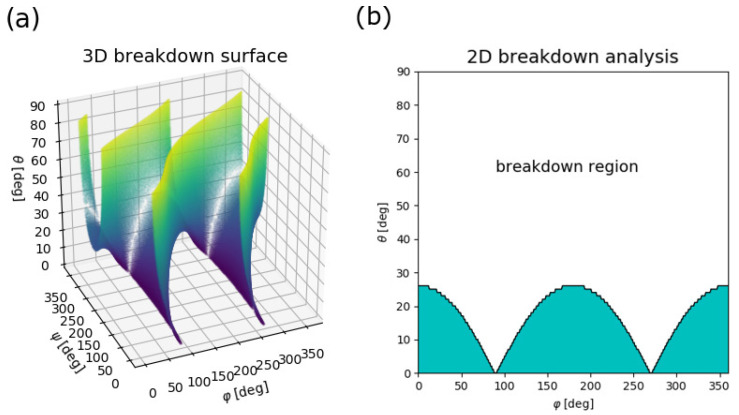
(**a**) Three-dimensional breakdown surface: the color code corresponds to the θ value purely for visualization. (**b**) Projection along the tilt direction ψ results in a 2D breakdown region and a sensing region (teal), where all $\psi \in {\left[0,360\right]}^{\circ }$ can be detected.

For a generic implementation, it is always possible to find a connected parameter space *P* that satisfies (2). One must only make sure that *P* does not cross a breakdown hypersurface, i.e., surfaces where Δp=0, as sketched in Figure 1 for two observables. Figure 3a displays such a breakdown surface for a system with ${\bar{r}}_{m}=\left(0,0,0\right)\;\mathrm{mm}$, $r_{s}=\left(3,0,0\right)\;\mathrm{mm}$ and and a magnetic moment $\mu_{0}\bar{m}=\left(0,1.25\cdot 10^{5},0\right)\;\mathrm{mT}\cdot {\mathrm{mm}}^{3}$ that corresponds to the magnetic moment of a cube having 5-mm-long sides and a 1000mT remanence field. Any feasible connected parameter space *P* must lie completely either above or below the breakdown surface. The limited choices for sensing regions for this specific implementation are immediately apparent: it is, for example, possible to realize a system addressing all tilt directions $\psi \in {\left[0,360\right]}^{\circ }$, but only when restricting tilt angle θ and rotation angle φ. The possibility to include all tilt directions can be understood through a projection of the breakdown surface along the ψ-direction: the 3D breakdown surface then becomes a 2D breakdown region which is shown in Figure 3b.

**Figure 4:**
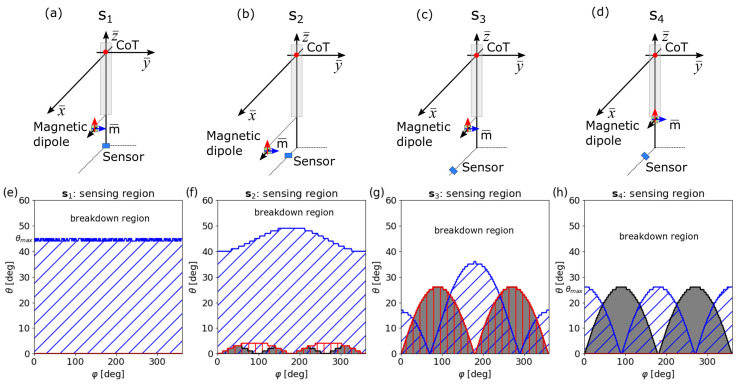
Sketches of magnetic joysticks together with the corresponding sensing regions: (**a**–**d**) geometric representations of implementations $s_{1}$,$s_{2}$, $s_{3}$, and $s_{4}$, respectively, and (**e**–**h**) the corresponding sensing regions. Black lines and grey regions are for $\bar{m}\|e_{x}$, blue is for $\bar{m}\|e_{y}$, and red is for $\bar{m}\|e_{z}$.

For a feasibility analysis including all angles, $\psi ,\varphi \in {\left[0,360\right]}^{\circ }$, magnet and sensor positions can be reduced to ${\bar{r}}_{m}=\left({\bar{x}}_{m},0,{\bar{z}}_{m}\right)$ and $r_{s}=\left(x_{s},0,z_{s}\right)$ with ${\bar{x}}_{m},x_{s}\geq 0$ without loss of generality due to the rotation symmetry. Fundamentally different behaviors are observed when the magnet displacement exceeds the sensor displacement (${\bar{x}}_{m}>x_{s}$) or when the opposite is the case (${\bar{x}}_{m}<x_{s}$). This is demonstrated in Figure 4, where four variations are displayed:

- system type $s_{1}:$ sensor in the center, magnet displaced ($x_{s}=0,{\bar{x}}_{m}>0$),
- system type $s_{2}:$ sensor and magnet displaced, magnet further out ($x_{s}<{\bar{x}}_{m}$),
- system type $s_{3}:$ sensor and magnet displaced, sensor further out ($x_{s}>{\bar{x}}_{m}$),
- system type $s_{4}:$ magnet in center, sensor displaced ($x_{s}>0,{\bar{x}}_{m}=0$).

Systems $s_{1}$ and $s_{4}$ are special cases of $s_{2}$ and $s_{3}$, respectively, with specific technical relevance.

In Figure 4e–h, the sensing regions for typical representatives of the four cases are shown: $s_{1}$ with ${\bar{r}}_{m}=\left(3,0,-3\right)$ and $r_{s}=\left(0,0,-6\right)$; $s_{2}$ with ${\bar{r}}_{m}=\left(3,0,-3\right)$ and $r_{s}=\left(0.5,0,-6\right)$; $s_{3}$ with ${\bar{r}}_{m}=\left(0.5,0,-3\right)$ and $r_{s}=\left(3,0,-6\right)$; and $s_{4}$ with ${\bar{r}}_{m}=\left(0,0,-3\right)$ and $r_{s}=\left(3,0,-6\right)$. For all the systems under investigation, gap and distance from the center of tilt are fixed to g=3 and $d_{CoT}=3$, respectively. All the positions are given in units of millimeters. Magnetic moment orientations along the three unit directions are considered for each system: $\bar{m}\|{\bar{e}}_{x}$ (black), $\bar{m}\|{\bar{e}}_{y}$ (blue), and $\bar{m}\|{\bar{e}}_{z}$ (red) with $\mu_{0}\left|m\right|=1.25\cdot 10^{5}\;\mathrm{mT}\cdot {\mathrm{mm}}^{3}$.

Figure 4 shows that the shape of the sensing region strongly depends on the implementation s. Specifically, the sensing region becomes maximal in size when the magnetic moment is perpendicular to the plane spanned by the sensor position and the lever axis for α=0 (blue sensing regions):

(11) $$m⊥(r_{s}\times e_{z}).$$

It is interesting to observe that full rotation $\varphi \in {\left[0,360\right]}^{\circ }$ can only be realized when the magnet displacement exceeds the sensor displacement (${\bar{x}}_{m}>x_{s}$) and while the perpendicularity condition (11) is simultaneously fulfilled. At the transition from ${\bar{x}}_{m}>x_{s}$ to ${\bar{x}}_{m}<x_{s}$ (see Figure 4f,g), the large connected sensing region splits up into two disconnected lobes with opposite signs of the Jacobian determinant.

**Figure 5:**
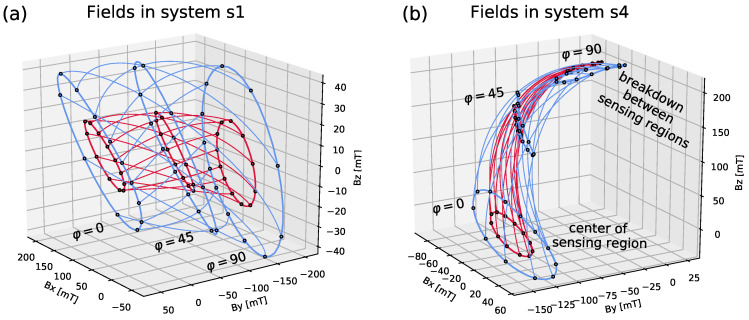
Magnetic field for the systems (**a**) $s_{1}$ and (**b**) $s_{4}$ illustrated in Figure 4: the fields are displayed for tilt angles $\theta =4^{\circ }$ (red) and $\theta =8^{\circ }$ (blue) for rotation angles $\varphi \in {\left[0,90\right]}^{\circ }$ and for 12 discrete tilt directions ranging from $\psi =0^{\circ }$ to $360^{\circ }$ in steps of $30^{\circ }$.

To better understand the breakdown for different implementations, the magnetic field of the two systems $s_{1}$ and $s_{4}$ is displayed in Figure 5. Different colors correspond to different tilt angles. The loops correspond to variations of the tilt direction $\psi \in {\left[0,360\right]}^{\circ }$, while the lines connecting different loops are variations of the rotation angle $\varphi \in {\left[0,90\right]}^{\circ }$. The iso-surfaces of $s_{1}$ systems are simple toroids, as shown in panel (a), while they cross each other at the transition from sensing region to breakdown region in $s_{4}$ type systems, which can be observed in panel (b). It is necessary to check if the displayed iso-surfaces form simply connected regions for a valid implementation; see the discussion in Appendix A.

Figure 5a shows that the tilt directions (displayed in steps of $30^{\circ }$) are not homogeneously spaced on the loops. For a fixed tilt angle and four fixed tilt directions separated by $90^{\circ }$, the rotation angle iso-lines form non-intersecting loops, i.e., circles all passing through each other without intersecting. This forms the basis of the *Mini-Drive* implementation [19,21], where only 4 discrete tilt directions are considered.

As a result of a systematic investigation, a general empirical rule is proposed to determine the limits of the sensing region for $s_{1}$ and $s_{4}$ under the assumption that the perpendicularity condition is fulfilled. The maximum tilt angle $\theta_{max}$ can be estimated as

(12) $$\theta_{\max}≃\arctan\left(|\frac{{\bar{x}}_{m}}{{\bar{z}}_{m}}|\right)+\arctan\left(|\frac{x_{s}}{z_{s}}|\right).$$

The values of $\theta_{\max}$ predicted by means of Equation (12) are reported in Figure 4e,h.

### 3.2. Quality Analysis

The feasibility analysis specifies which systems are in principle possible but provides no information about the quality, which is expressed in (4) by the DOS and the state separation Δp. A specific implementation s is considered to be of high quality when all its mechanical states α∈P are well-separated in magnetic space, as expressed by Equation (5), which ensures easy state identification by the sensor.

**Figure 6:**
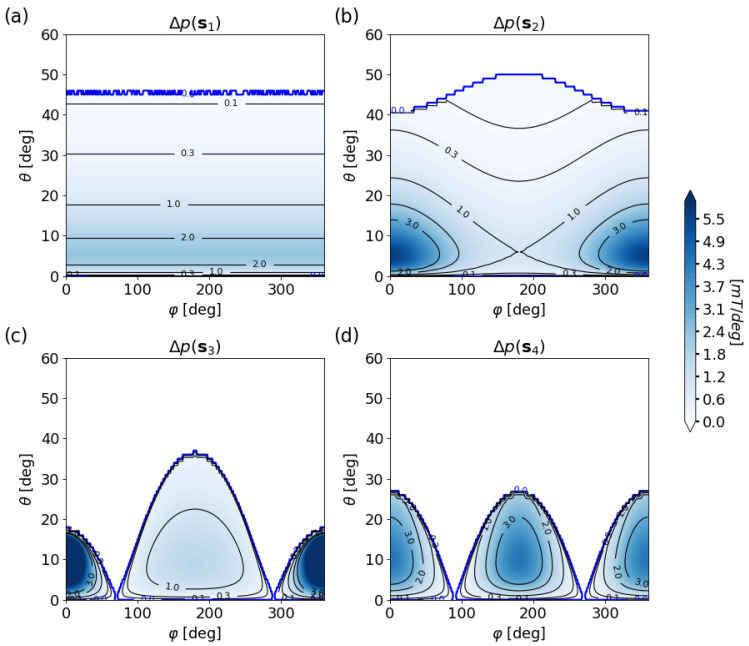
(**a**–**d**) State separation Δp for the four different implementations $s_{1}$ to $s_{4}$ with $\bar{m}\|{\bar{e}}_{y}$: the thick blue contour delimits the sensing region.

For a further analysis of possible implementations, the state separations of the system types $s_{1},s_{2},s_{3},s_{4}$ defined in Section 3.1 are computed, assuming that the perpendicularity condition (11) is valid. The surprising results are shown in Figure 6a–d, where the sensing region is delimited in blue (in accordance with Figure 4) and the shading corresponds to the state separation.

All systems exhibit low state separations for very small tilt angles. This is the result of the chosen coordinate representation, where the mechanical state density tends to infinity when θ→0. However, the singularity is typically avoided by the dead-band $\theta_{\mathrm{dead}}$ of several degrees, which typically limits position computation to $\theta >\theta_{\mathrm{dead}}$ in most applications [13].

When the sensor lies in the center and the magnet is displaced (system $s_{1}$), the rotation symmetry makes the DOS independent of the rotation angle φ, which is optimal for the realization of $360^{\circ }$ rotation. However, the state separation decays quickly for increasing tilt angles, as the distance between magnet and sensor increases, which makes it difficult to exploit the large $\theta_{\max}$ provided by the feasibility study above.

For small sensor displacement, the system makes a transition to $s_{2}$. The state separation then becomes inhomogeneous in the rotation direction. Rotation angles about $\varphi =0^{\circ }$ can be resolved better at the expense of a lower state separation at $\varphi =180^{\circ }$. This is opposite to the variation of $\theta_{\max}$, which becomes smaller around $\varphi =0^{\circ }$ but increases at $\varphi =180^{\circ }$.

When the sensor and magnet are at the same position, there is only a single feasibility lobe with maximum at $\varphi =180^{\circ }$ and complete breakdown at $\varphi =0^{\circ }$. Then, by displacing the sensor beyond the magnet, system $s_{3}$ is realized. The large central lobe reduces in size and a second small lobe appears at $\varphi =0^{\circ }$. Unfortunately, the large lobe—which is good, from a feasibility point of view, for realizing large ranges of the parameters of interest—features a low state separation, while the small lobe exhibits a large one. Both lobes become of equal size with similar state separation when the magnet is located in the center, thereby realizing the system $s_{4}$.

In summary, an optimal system requires large state separation in the parameter region of interest. Small and inhomogeneous state separations reduce the quality of sensing regions. As shown by the above study, the low quality of the large lobe in $s_{3}$ and the inhomogeneity of the state separation in system $s_{2}$ make these implementations inferior in a technical sense to the implementations $s_{1}$ and $s_{4}$.

### 3.3. Optimized Systems with Cuboid Magnets

The two most relevant system types $s_{1}$ and $s_{4}$, introduced in Section 3.1, are here optimized in accordance with Equation (6) using the realistic parameters and finite-sized magnets outlined in Section 2.3. The goal of the optimization is to determine the best possible set of realistic system parameters $s_{\mathrm{opt}}$ for given observable ranges of interest.

In both systems, the perpendicularity condition (11) is satisfied, $\bar{M}=\left(0,1000,0\right)\;\mathrm{mT}$, and an off-the-shelf cubical magnet with a=b=c=5mm is used. For $s_{1}$ optimization, the sensor is fixed in the center ($x_{s}=0\;\mathrm{mm}$), which results in a state density independent of the rotation angle; see Figure 6a. The parameter space of interest is chosen as $\psi \in {\left[0,360\right]}^{\circ }$, $\theta \in {\left[2,12\right]}^{\circ }$ and complete rotation $\varphi \in {\left[0,360\right]}^{\circ }$. For $s_{4}$ optimization, the magnet is constrained to the center (${\bar{x}}_{m}=0\;\mathrm{mm}$) and the parameter space of interest is chosen as $\psi \in {\left[0,360\right]}^{\circ }$, $\theta \in {\left[2,12\right]}^{\circ }$ and $\varphi \in {\left[-30,30\right]}^{\circ }$. For both configurations, a common dead-band $\theta_{\mathrm{dead}}=2^{\circ }$ is taken into account to avoid the singularity in the state separation at $\theta =0^{\circ }$. Given that most magnetic joystick systems employed in technological applications are millimeter sized, in the optimization procedure, the following reasonable boundaries are used for both the implementations: g∈[2,4]mm and $d_{\mathrm{CoT}}\in \left[3,5\right]\;\mathrm{mm}$. In addition, the lateral displacements are chosen as ${\bar{x}}_{m}\in \left[0.1,15\right]\;\mathrm{mm}$ for $s_{1}$ and $x_{s}\in \left[0.1,15\right]\;\mathrm{mm}$ for $s_{4}$.

As expected, for the two optimized systems $s_{1,\mathrm{opt}}$ and $s_{4,\mathrm{opt}}$ the optimization procedure yields the minimum allowed value for the gap g=2mm and the maximum value of the distance of the magnet from the center of tilt $d_{\mathrm{CoT}}=4.97\;\mathrm{mm}$: these correspond to small magnet-sensor distances and maximal mechanical state separation. The remaining displacements lead to a maximal state separation when ${\bar{x}}_{m}=1.52\;\mathrm{mm}$ and $x_{s}=3.26\;\mathrm{mm}$ for $s_{1,\mathrm{opt}}$ and $s_{4,\mathrm{opt}}$, respectively.

**Figure 7:**
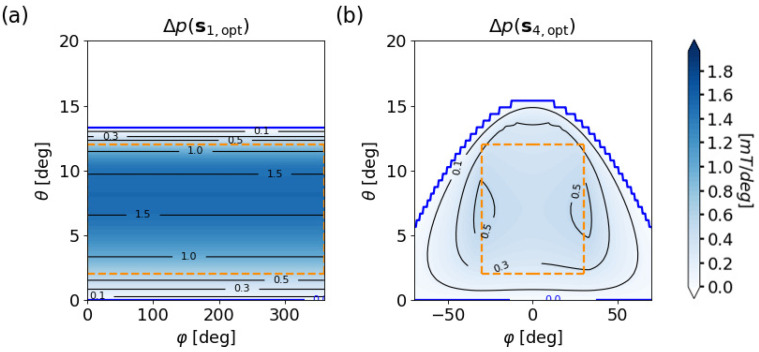
State separation Δp of the optimized systems (**a**) $s_{1,\mathrm{opt}}$ and (**b**) $s_{4,\mathrm{opt}}$ with $\bar{m}\|{\bar{e}}_{y}$. Orange dashed lines outline the parameter space α.

Figure 7 displays the state separation Δp for the optimal systems.

Panels (a) and (b) show that $\Delta p(s_{1,\mathrm{opt}})$ is generally larger than $\Delta p(s_{4,\mathrm{opt}})$ for these implementations with quality factors, computed via Equation (5), of $Q(s_{1,\mathrm{opt}})=$ 0.64 mT and $Q(s_{4,\mathrm{opt}})=$ 0.23 mT, respectively. The lower quality of $s_{4,\mathrm{opt}}$ is a result of the much larger lateral displacement which leads to a greater distance between magnet and sensor.

### 3.4. Experimental Results

In this section, the theoretical predictions are tested in an experiment realizing a system of type $s_{4}$ with observables of interest $\theta \in {\left[2,15\right]}^{\circ }$ and $\varphi \in {\left[-30,30\right]}^{\circ }$ in accordance with the nautical device presented in [13]. The $s_{4}$ configuration was chosen because, in contrast to $s_{1}$ systems, the symmetry allows for integration of two sensors in the same plane (i.e., on a single printed circuit board (PCB)), each one in a different sensing lobe. The second sensor can be used both for redundancy reasons and for the need to compensate external magnetic stray fields by evaluating a differential signal.

The chosen system parameters are the following: a cubical magnet with side length 5mm, an airgap of g=2mm, a magnet distance to the center of tilt of $d_{\mathrm{CoT}}=6.83\;\mathrm{mm}$, and a sensor displacement of $x_{s}=\pm 5.52\;\mathrm{mm}$. These parameters are selected as a result of optimization, as outlined in Section 3.3. This optimization includes potential large system fabrication tolerances ($\delta {\bar{x}}_{m}=\pm 0.5\;\mathrm{mm}$, $\delta {\bar{y}}_{m}=\pm 0.5\;\mathrm{mm}$, $\delta {\bar{z}}_{m}=\pm 0.5\;\mathrm{mm}$, $\delta x_{s}=\pm 0.25\;\mathrm{mm}$, $\delta y_{s}=\pm 0.25\;\mathrm{mm}$, and δg=±0.5mm) to ensure reliability and stability of the mechanical system.

**Figure 8:**
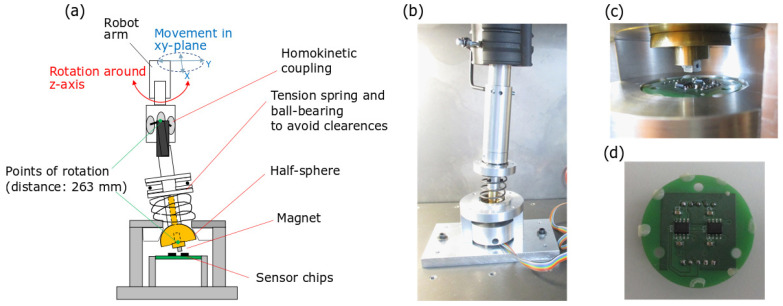
Experimental setup: (**a**) schematic of the setup; (**b**) photo of the mechanical setup; (**c**) zoom-in on the center ball, magnet, and PCB with sensors; and (**d**) top view of the PCB.

The experimental setup is outlined in Figure 8. A custom three-axis joystick is coupled to a robot arm to realize precise mechanical states. The chosen four-axis pick-and-place robot is commonly used to examine linear position and angle sensors. The robot arm is parallel to the z-axis and can move linearly to any point in x-y-z-space with a precision of 30 μm. A rotation of the arm around the z-axis is possible with a precision of $0.02^{\circ }$. The joystick lever is connected to the robot arm with the help of homokinetic coupling with an integrated length compensation element. This configuration overcomes several difficulties that arise from coupling the linear robot motion to the spherical joystick motion, including a necessary robot arm length compensation, a nonlinear connection between joystick rotation and robot rotation as well as a nonuniform transmission of torque which is not ideal to account for clearances in the setup. Further details on this kind of couplings are reported in [48]. The expected positioning error in this implementation is $0.01^{\circ }$ in tilt and $0.02^{\circ }$ in rotation when clearances are neglected.

The joystick itself is realized through a center ball (half sphere) which is connected to the rod and pressed into a spherical cavity by two springs and a ball-bearing in order to minimize further clearances in the setup. Figure 8a shows a sketch of the setup, whereas a picture of the actual mechanical realization is displayed in Figure 8b.

The magnet is fixed to the center ball, while two 3D sensors are mounted on a PCB and integrated into the system along the positive and negative directions of the *x*-axis; see Figure 8c,d. To account for the low state separation, a high-precision 3D Hall sensor with a resolution of 8μT is chosen. The sensor is configured in such way that it provides raw values for the three components of the magnetic flux density.

**Figure 9:**
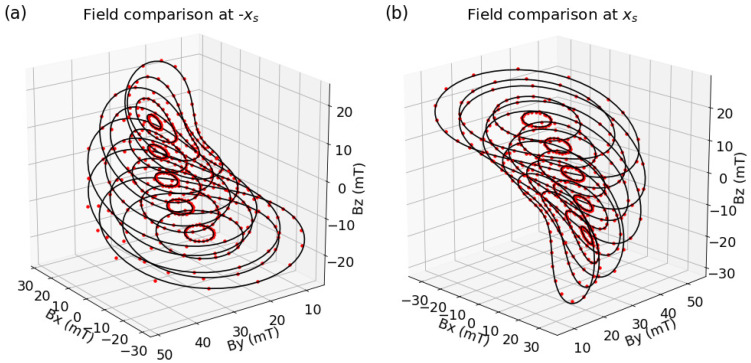
Comparison between experimental measurements (red dots) and analytical calculation (black lines) in an $s_{4}$-type system with two sensors located at $x_{s}=-5.5\;\mathrm{mm}$ (**a**) and $x_{s}=5.5\;\mathrm{mm}$ (**b**), respectively.

In the experiment 16,200 mechanical positions are measured in an angle grid with step sizes $\Delta \psi =8^{\circ }$, $\Delta \theta =1^{\circ }$ and $\Delta \varphi =2.5^{\circ }$. A comparison between experimental data and theoretical predictions (simulated as discussed in Section 2.4) is shown in Figure 9 for the two sensors at $x_{s}=5.5\;\mathrm{mm}$ and $x_{s}=-5.5\;\mathrm{mm}$.

The theoretical values show a mean deviation from experimental measurements by 0.1 mT for both the sensors in $x_{s}=-5.5\;\mathrm{mm}$ and $x_{s}=5.5\;\mathrm{mm}$. The high consistency between theory and experiment is achieved by fitting the theoretical predictions onto the experimental data by variation of 27 tolerances that include sensor position, orientation, gains and offsets, magnet position, orientation, dimensions, magnetization, and experimental angle offsets. All computed tolerances lie within reasonable ranges. The resulting mean angles errors are $\langle e_{\theta }\rangle =0.067^{\circ }$, $\langle e_{\varphi }\rangle =0.126^{\circ }$ and $\langle e_{\psi }\rangle =0.491^{\circ }$ and at the 99 percentile, the maximum errors are $e_{\theta ,max}=0.481^{\circ }$, $e_{\varphi ,max}=0.658^{\circ }$ and $e_{\psi ,max}=2.278^{\circ }$ for single sensor evaluation. Such errors are at the same level with literature values of 3-DoF and 6-DoF motion tracking [8] where, however, multiple sensors are used for read-out.

## 4. Conclusions

In this work, the difficulties related to magnet position and orientation (MPO) system design are discussed. A new method for the computation of MPO system layouts is proposed, aimed at maximizing the state separation through a global optimization procedure that is enabled by computationally fast analytical solutions of permanent magnet problems. A comparison to sophisticated topology optimization shows that the proposed computationally inexpensive ansatz can achieve excellent results.

The formalism is applied to study the three-axis-joystick problem. It is shown for the first time that continuous three-axis motion tracking can be realized with only a single magnet and a single 3D magnetic field sensor when fulfilling a specific design criterion that relies on perpendicularity between rotation axis, magnet or sensor displacement, and magnetization direction. For realistic cubical magnets with 5 mm sides and 1000 mT remanence field, a full $360^{\circ }$ rotation and tilts up to $12^{\circ }$ can be realized at 2 mm airgap with state separation close to 1 mT/${}^{\circ }$. Much larger tilt angles beyond $60^{\circ }$ can also be achieved at the expense of state separation. The proposed systems allow for three-axis-joystick motion tracking at unparalleled cost-efficiency. The computations are confirmed by an experimental study where the measured fields are consistent with the theoretical predictions, and resulting mean angle errors are below $0.5^{\circ }$.

This study demonstrates the potential of the proposed formalism in designing novel MPO systems. Future work is dedicated to include system fabrication tolerances in the design process as well as efficient calibration, inversion strategies, and stray field compensation.

## Figures and Tables

**Figure A1:**
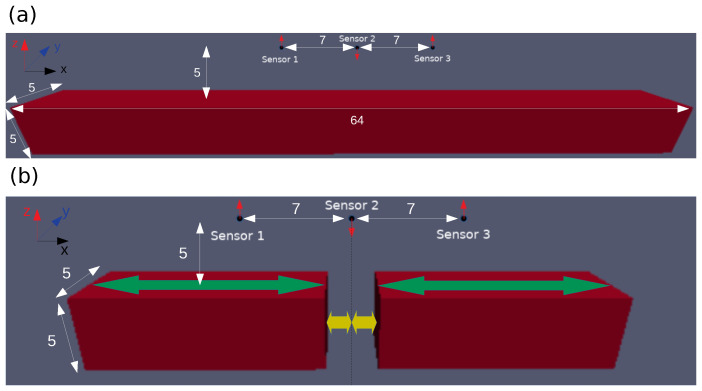
Illustration of the dimensions and the initial degrees of freedom (DoFs) for (**a**) topology optimization (magnetic material separated in cubic cells with edge length 0.2 within the red target area) and (**b**) the shape variation method (length of magnets and distance in-between, marked with green and yellow arrows, respectively).

**Figure A2:**
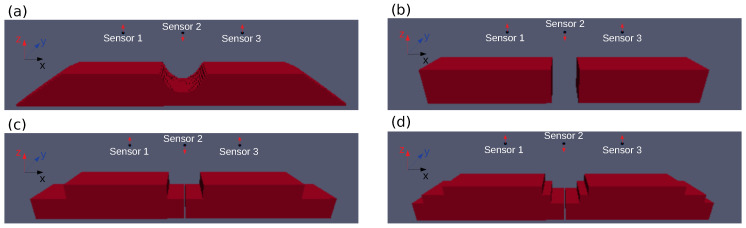
Results of optimization via the adjoint method (**a**) and the shape variation with one (**b**), two (**c**), and three (**d**) magnetic layers.

**Figure A3:**
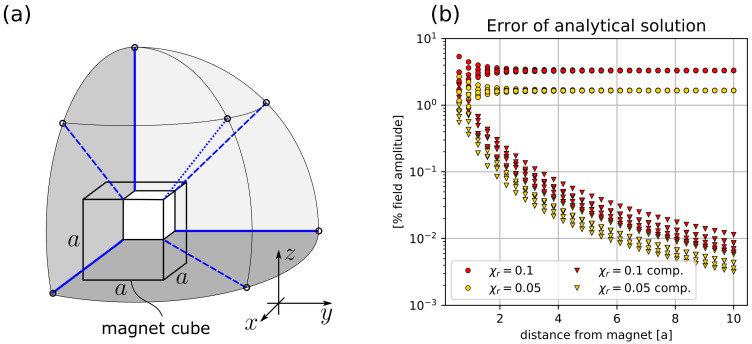
(**a**) Sketch of representative positions (blue lines) where the field is evaluated and (**b**) relative error of the analytical solution at those positions.

## Abbreviations

The following abbreviations are used in this manuscript:

MPO	Magnetic position and orientation
DoF	Degree(s) of freedom
ABS	Anti-lock braking system
CCTV	Closed-circuit television
DOS	Density of states
LCS	Local coordinate system
FE	Finite element
DE	Differential evolution
PCB	Printed circuit board

## Appendix A. Connectedness Requirement

The requirement for $B_{\mathrm{space}}$ to be simply connected suppresses a potential periodicity of the sensor output that would render the one-to-one correspondence between mechanical states *P* and sensor outputs $B_{\mathrm{space}}$ invalid.

This is best demonstrated with an example. Consider a quadrupole magnet disc that is used in an end-of-shaft application [6,43]. The shaft angle $\varphi \in {\left[0,360\right]}^{\circ }$ should be determined by a 2D magnetic field sensor located above the disc. The sensor outputs are then $B=B_{0}\left(\cos\left(2\varphi \right),\sin\left(2\varphi \right)\right)$. In this case, $J^{T}J=4B_{0}^{2}$ is computed so that Equation (3) always holds true. The sensor output is also always smooth; however, it is periodic with $180^{\circ }$ so that a one-to-one correspondence is not possible for all rotations. Such a periodicity, which makes the chosen implementation invalid, becomes immediately visible by checking the iso-surfaces of $B_{\mathrm{space}}$ (as outlined in Section 3.1).

## Appendix B. Field Shaping with Topology Optimization (Adjoint Method) and Shape Variation

Magnetic field shaping relates to optimization problems for designing magnet systems that are able to generate given target fields $B^{\mathrm{target}}$. Therefore, the error $d(B,B^{\mathrm{target}})$ is minimized with respect to a suitable chosen deviation measure d(·,·). Using the formalism introduced in Section 2.1, this can be expressed through the quality factor $Q\left(s\right):=-d\left(B,B^{\mathrm{target}}\right)$, which has to be maximized with respect to the system design parameters s∈S. In this appendix, two different fundamental approaches for magnetic field shaping are compared.

In the very general context of topology optimization, a target region Ω for the magnets is specified. An indicator function ρ:Ω⟶[0,1] defines the magnetic material density within this region, where 0 means no and 1 is full magnetic material at the position. In this case, the system parameter space *S* consists of all possible indicator functions ρ. In practice, the infinite function space *S* is reduced in dimension using an FE or finite difference approach and the final DoF depends on the chosen discretization. Since *S* might still be very large, non-gradient-based optimization methods are mostly inefficient. The sophisticate approach of the adjoint method [24,30,31,32] provides a computationally cheap possibility to calculate the gradient of the target function *Q*. However, necessary differentiability properties also restrict the possible deviation measures d(·,·).

In contrast to topology optimization, the shape variation method makes use of a parametrization of the magnetic regions. Depending on the specific problem and requirement of the solutions, a reasonable choice of shape parameters may lead to far less DoFs compared to the adjoint method approach. This reduces the dimension of the system parameter space *S* and allows also non-gradient-based optimization procedures, like the DE algorithm [45] discussed in Section 3.3.

A simple optimization example is presented to demonstrate the two approaches and to discuss their differences. Three 1D magnetic sensors that measure the *z*-component of the field are placed at positions $r_{1}=\left(-7,0,0\right),r_{2}=\left(0,0,0\right)$ and $r_{3}=\left(7,0,0\right)$. The goal is to maximize the field at the outer positions $r_{1},r_{3}$ and to minimize it at the centered position $r_{2}$. The target field $B_{\mathrm{target}}$ can therefore formally be defined as $B_{z}^{\mathrm{target}}\left(r_{1}\right)=B_{z}^{\mathrm{target}}\left(r_{3}\right)=\left(0,0,\infty \right)$ and $B_{z}^{\mathrm{target}}\left(r_{2}\right)=\left(0,0,0\right)$. To measure the “distance to infinity” of the *z*-component in a suitable way, we could define the deviation measure for instance as

(A1) $$d\left(B,B^{\mathrm{target}}\right):=\sum_{i}w_{i}\cdot {\left(B_{z}\left(r_{i}\right)-B_{z}^{\mathrm{target}}\left(r_{i}\right)\right)}^{2},$$

with weights $w_{i}\geq 0,\sum_{i}w_{i}=1$, where we additionally set

(A2) $${\left(B_{z}\left(r_{i}\right)-B_{z}^{\mathrm{target}}\left(r_{i}\right)\right)}^{2}:=-B_{z}^{2}\left(r_{i}\right)\;\;\;\;\mathrm{if}\;B_{z}^{\mathrm{target}}\left(r_{i}\right)=\infty ,$$

since increasing values of $B_{z}^{2}$ shall decrease the distance to $B^{\mathrm{target}}$ in that case. In that example, we choose $w_{1}=w_{3}=0.4,w_{2}=0.2$ and obtain the function

(A3) $$Q\left(s\right)=-d\left(B,B^{\mathrm{target}}\right)=0.4\cdot B_{z}{\left(r_{1}\right)}^{2}-0.2\cdot B_{z}{\left(r_{2}\right)}^{2}+0.4\cdot B_{z}{\left(r_{3}\right)}^{2},$$

which favors strong fields at the left and the right sensors and weak fields in the middle. Figure A1a illustrates the target region for the adjoint method, which is divided into 320×25×25 cubic cells, leading to $2\times 10^{5}$ DoF. For simplification, ρ is chosen as a binary function [49] with output in {0,1}. Under consideration of the specific alignment and symmetry of the problem, shape variation can be performed with only two DoFs, namely the length of two magnets and the distance between them (see Figure A1b). All length dimensions, magnetization, and fields are understood in arbitrary units and do not affect the results.

The resulting geometries of the two optimizations are shown in Figure A2. The large number of DoFs of the adjoint method yields a special magnet geometry, which is the “best possible” magnet shape $s_{\mathrm{opt}}$ when neglecting the finite discretization. Shape variation, on the other hand, simply places the two cuboid magnets with optimal length at the optimal distance from each other. While the two solutions seem geometrically very different from one other, the performance of the simple shape variation is only 3.35% below the optimal solution maximizing the cost function (A3).

In addition, shape variation is easily extended to more DoFs by combining multiple magnets. Here, cuboid stacks are tested and each additional layer adds three more DoFs (length, *x*-separation, and height of the new magnet pair). The result of such an optimization is displayed in Figure A2c,d for two and three layers of magnets, respectively.

Deviations of the optimization results with respect to the optimal solution are reported in Table A1.

**Table A1:** Deviation of the optimum quality factor Q(**s**) defined in Equation (A3) for the shape variation with various number of magnet layers, corresponding to Figure A2b–d: values are reported in % compared to the solution of the adjoint method in Figure A2a.

Layers	DoF	Deviation from $Q(s_{\mathrm{opt}})$ [%]
1	2	3.35
2	5	0.79
3	8	0.49

It can be seen that shape variation performs surprisingly well, reaching more than 99% of the possible quality in the presented optimization problem, despite the crude geometric approximation when compared to the optimal solution. This discrepancy is expected to become even smaller by increasing the distance from the magnet target region. For many applications, such a small gain might not justify the additional effort to realize the complex optimal magnet shape. While the presented problem is reminiscent of MPO systems, it is also of a very simple nature and it must be pointed out that no general conclusion should be drawn. A reasonable summary of the findings of this section is given in Table A2.

**Table A2:** Overview of the comparison between optimization with topology optimization and shape variation.

	Topology Optimization	Shape Variation
number DoF	large	small
optimization algorithm	gradient based (local)	function evaluation based (global)
quality factor	requires derivation	requires fast evaluation
optimum (magnet shape)	very accurate, but possibly local	inaccurate, depending on choice of DoF
optimum (magnetic field)	very accurate, but possibly local	possibly very accurate, depending on DoF choice
application case	theoretical optimum solutions	good practical solutions

## Appendix C. Computation of the Rotation Matrix

Here, the rotation matrices used to track the motion of the magnet are given explicitly. To this aim, it is convenient to split the three-axis motion in two independent (non-commutative) rotation operations. First, the lever—and with it, the magnet—is rotated about the *z*-axis by the angle φ expressed via the rotation matrix $R_{1}\left(\varphi \right)$:

(A4) $$R_{1}\left(\varphi \right)=\left(\begin{array}{ccc} \cos\varphi & -\sin\varphi & 0\\ \sin\varphi & \cos\varphi & 0\\ 0 & 0 & 1 \end{array}\right).$$

Then, for the application of the tilt, the lever is rotated by an angle θ about an axis (cos(ψ),sin(ψ),0) that passes through the center of tilt in the origin of the global coordinate system. Such an angle-axis rotation is mathematically formulated by means of the quaternion notation. The unitary quaternion u representing the rotation of an angle θ about the axis $u_{i}$ can be written as $u=\left(\cos\left(\frac{\theta }{2}\right),u_{1}\sin\left(\frac{\theta }{2}\right),u_{2}\sin\left(\frac{\theta }{2}\right),u_{3}\sin\left(\frac{\theta }{2}\right)\right)$. The components of the rotation axis $\left(u_{1},u_{2},u_{3}\right)$ are calculated by means of the spherical coordinates transformation: $u_{i}$$=\left(\sin(\pi /2)\cos\psi ,\sin(\pi /2)\sin\psi ,\cos(\pi /2)\right)$, where ψ is the azimuth angle and $\frac{\pi }{2}$ is the polar angle. Hence, the explicit expression of the unitary quaternion takes form: $u=\left(\cos\left(\frac{\theta }{2}\right),\cos\left(\psi \right)\sin\left(\frac{\theta }{2}\right),\sin\left(\psi \right)\sin\left(\frac{\theta }{2}\right),0\right)$ and the corresponding rotation matrix is

(A5) $$R_{2}\left(\theta ,\psi \right)=\left(\begin{array}{ccc} \cos^{2}\left(\frac{\theta }{2}\right)+\cos2\psi \sin^{2}\left(\frac{\theta }{2}\right) & \sin^{2}\left(\frac{\theta }{2}\right)\sin2\psi & \sin\psi \sin\theta \\ \sin2\psi \cos^{2}\left(\frac{\theta }{2}\right) & \cos^{2}\left(\frac{\theta }{2}\right)-\cos2\psi \sin^{2}\left(\frac{\theta }{2}\right) & -\cos\psi \sin\theta \\ -\sin\psi \sin\theta & \cos\psi \sin\theta & \cos\theta \end{array}\right).$$

Finally, the full three-axis motion is described via $R\left(\alpha \right)=R_{2}\left(\theta ,\psi \right)R_{1}\left(\varphi \right)$. Such a matrix is used in Equations (7), (8), and (9) to compute the magnet position $r_{m}\left(\alpha ,s\right)$, orientation $e_{i}^{m}\left(\alpha ,s\right)$ and the magnetic field B(α,s) of a given implementation s in the LCS. Eventually, R(α) is given in full by

(A6) $$R\left(\alpha \right)=\left(\begin{array}{ccc} \cos^{2}\left(\frac{\theta }{2}\right)\cos\varphi +\cos\left(\varphi -2\psi \right)\sin^{2}\left(\frac{\theta }{2}\right) & -\cos^{2}\left(\frac{\theta }{2}\right)\sin\varphi -\sin^{2}\left(\frac{\theta }{2}\right)\sin\left(\varphi -2\psi \right) & \sin\psi \sin\theta \\ \sin\psi \cos^{2}\left(\frac{\theta }{2}\right)-\sin^{2}\left(\frac{\theta }{2}\right)\sin\left(\varphi -2\psi \right) & \cos^{2}\left(\frac{\theta }{2}\right)\cos\varphi -\cos\left(\varphi -2\psi \right)\sin^{2}\left(\frac{\theta }{2}\right) & -\cos\psi \sin\theta \\ \sin(\psi -\varphi )\sin\theta & \cos(\psi -\varphi )\sin\theta & \cos\theta \end{array}\right).$$

## Appendix D. Magpylib Code

Magpylib is a publicly developed, open-source Python package dedicated to magnetic field computation in MPO systems [41]. The central ambition of Magpylib is to provide magnetic field computation and geometric source manipulation with maximal simplicity. A program for computing the output of a 3D magnetic field sensor in the proposed three-axis-joystick system requires only few lines of code:

Lines 1–3 are library imports. Lines 5–8 define the system parameters ${\bar{r}}_{m}=\left(2,0,-4\right)\;\mathrm{mm}$ and $r_{s}=\left(0,0,-8\right)\;\mathrm{mm}$ and a designated set of computation angles $\alpha ={\left(11,22,33\right)}^{\circ }$. In line 11, an instance of the cuboid magnet class *magnet.Box* is created with $\mu_{0}\bar{M}=\left(0,1000,0\right)\;\mathrm{mT}$ and dimension (a,b,c)=(3,3,3)mm. Lines 13–18 apply the rotation and tilt operations transforming from local to global coordinates. The rotation is applied using quaternion (angle-axis) representation. In line 21, the field is computed at the sensor position, which is then printed in line 24.

## Appendix E. Demagnetization

The dipole field, see Equation (10), corresponds to the field generated by the delta response $M\left(r\right)=M_{0}\delta \left(r\right)$, so that the magnetic field of an arbitrary magnetization distribution M(r) is expressed through the integral [38],

(A7) $$H\left(r\right)=\frac{1}{4\pi }\int \frac{\left(r-r^{\prime }\right)\left(M\left(r^{\prime }\right)\cdot \left(r-r^{\prime }\right)\right)}{|r-r^{\prime }{|}^{5}}-\frac{M(r^{\prime })}{|r-r^{\prime }{|}^{3}}dr^{\prime }.$$

For uniform permanent magnets with constant magnetization $M_{0}$, Equation (A7) can be integrated directly and brought to an analytic expression or even to a closed form for simple geometric magnet shapes like cuboids [39,40], cylindrical geometries [50,51], or facet bodies [52]. Based thereon, the field can be obtained with little computational effort.

However, realistic materials are subject to demagnetization, meaning that the magnetization M at position r depends on the field H at this position, which is described by a material response M(H). As a result, the magnetization of an initially uniform magnetized body reduces and becomes inhomogeneous through self-interaction or interaction with external fields. State-of-the-art magnets are uniformly magnetized in a Helmholtz field so that demagnetization renders the analytic formulas in principle invalid. In the following, accuracy of the analytical formulas is tested when comparing with magnets having a realistic material response. The simplest material law for permanent magnets is given by

(A8) $$M\left(H\right)=M_{0}+\chi_{r}H,$$

which corresponds to a linearized hysteresis curve about an imprinted remanence magnetization $M_{0}$. The remanent susceptibility $\chi_{r}$ describes a linear material response to the field H. Modern high-grade SmCo, NdFeB, or ferrite materials feature extremely large coercive fields [53] and small demagnetization slopes as low as $\chi_{r}>0.05$, as found in the product portfolio of any permanent magnet manufacturer. As a consequence, Equation (A8) is very accurate and demagnetization effects are expected to be small. A method of moments code [54] is implemented to determine the demagnetization effects of a cubic magnet with $M_{0}\|e_{z}$. For a chosen discretization of the cube into 6859 ($19^{3}$) cells, the relative error of the magnetic field of the MOM computation easily undercuts $10^{-4}$ in this study, as was determined by an additional FE simulation (ANSYS Maxwell).

The simulation results are displayed in Figure A3. The field is sampled along several representative lines (blue): perpendicular to the magnet surfaces (solid lines), perpendicular to the edges (dashed lines), and outwards from the corner (dotted line) in the first octant defined by the cube. The relative error along these lines is shown (in percent of the field amplitude) in Figure A3b for two different susceptibility values $\chi_{r}=0.1$ (red) and $\chi_{r}=0.05$ (yellow). The seven different lines are represented by multiple markers at each distance.

Without additional compensation, demagnetization effects result in an error up to a few percent of the field amplitude (circles). However, this error is mostly of a quantitative nature and can be analytically compensated simply by correcting the total dipole moment. The correction factors are $M/M_{0}\approx 0.9679$ for $\chi_{r}=0.1$ and $M/M_{0}\approx 0.9837$ for $\chi_{r}=0.05$. An approximation of the correction factors can also be computed analytically [55]. The relative error of the compensated analytical solution (triangles) decays quickly with the distance from the magnet as the cube field starts to approach the form of a dipole field.

Variation problems that include the magnetization amplitude (see, e.g., the fitting in Section 3.4) automatically draw he high accuracy of the compensated analytical solution, but even the few percent errors associated with the uncompensated solution are typically acceptable for magnet system design.
